# An Experimental Generator for Production of High-Purity ^212^Pb for Use in Radiopharmaceuticals

**DOI:** 10.2967/jnumed.122.264009

**Published:** 2023-01

**Authors:** Ruth Gong Li, Vilde Yuli Stenberg, Roy Hartvig Larsen

**Affiliations:** 1 Institute of Clinical Medicine, University of Oslo, Oslo, Norway;; 2 Department of Radiation Biology, Institute of Cancer Research, Norwegian Radium Hospital, Oslo University Hospital, Oslo, Norway;; 3 Oncoinvent AS, Oslo, Norway;; 4 ArtBio AS, Oslo, Norway; and; 5 Sciencons AS, Oslo, Norway

**Keywords:** lead-212, ^212^Pb, ^220^Rn, ^212^Pb generator, radionuclide production

## Abstract

The feasibility, performance, and radiation safety of an experimental generator were evaluated to efficiently produce ^212^Pb intended for radiopharmaceuticals. **Methods:** The generator consisted of a flask with a removable cap containing a source of ^224^Ra or ^228^Th absorbed on quartz wool. Gaseous ^220^Rn emanated from the decaying source, which subsequently decayed to ^212^Pb, which was adsorbed on the flask’s interior surface. The ^212^Pb was collected by washing the flask with 0.5–1 mL of 0.1 M HCl. **Results:** The generator collector flask trapped 62%–68% of the ^212^Pb, of which more than 87% (tested up to 26 MBq) could be harvested. The obtained ^212^Pb solution had a high purity (>99.98%) and could be used for the preparation of radioconjugates with more than 97% radiochemical purity. Future designs of the generator should aim to further reduce the risk of radon and γ-energy exposure to operators. **Conclusion:** The presented technology is a promising method for easy and convenient ^212^Pb production.

## Visual Abstract

**Figure:**
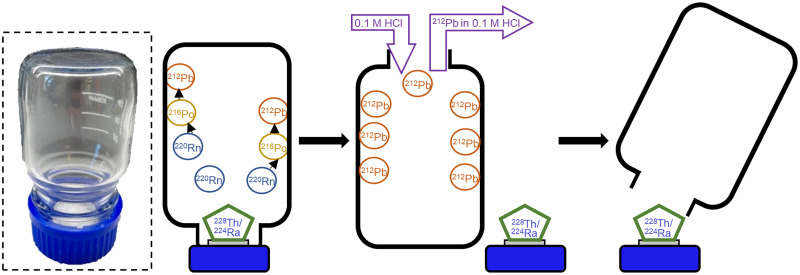

Lead-212 (^212^Pb; half-life, 10.6 h), a β-emitter itself, is an in vivo generator of α-particles through the α-emitting progenies ^212^Bi and ^212^Po. Convenient chelation chemistry makes ^212^Pb suitable for targeted α-therapy (1). However, the radiolabeling of targeting agents should preferably be performed on-site because of the short half-life of ^212^Pb. Rapid and efficient processes are required to ensure sufficient ^212^Pb availability for end users.

As a member of the thorium series (Fig. 1), ^212^Pb can be obtained from generators that contain the longer-lived mother nuclides ^228^Th (half-life, 1.9 y) or ^224^Ra (half-life, 3.6 d). Current generators are based on isolating ^212^Pb from ^224^Ra or ^228^Th through several purification steps. ^224^Ra has become the preferred radionuclide source over ^228^Th to minimize radiation hazards (1). A generator used to supply ^212^Pb for clinical trials by Orano Med is based on ^224^Ra immobilized on a cation-exchange column from which ^212^Pb can be eluted (2*–*4). The eluate is then evaporated and treated several times with concentrated acid before the final solution is ready for radiolabeling (3). A similar generator purchased from Oak Ridge National Laboratory was integrated into an automated synthesis module, where ^212^Pb was eluted in dilute HCl for labeling of peptides (5). An alternative method that avoids purification of ^212^Pb from the generator source material uses a solution of ^224^Ra/^212^Pb in equilibrium directly for the radiolabeling process (6*,*^7^). However, this procedure still requires a final purification step to remove ^224^Ra and unconjugated daughters.

**FIGURE 1.**
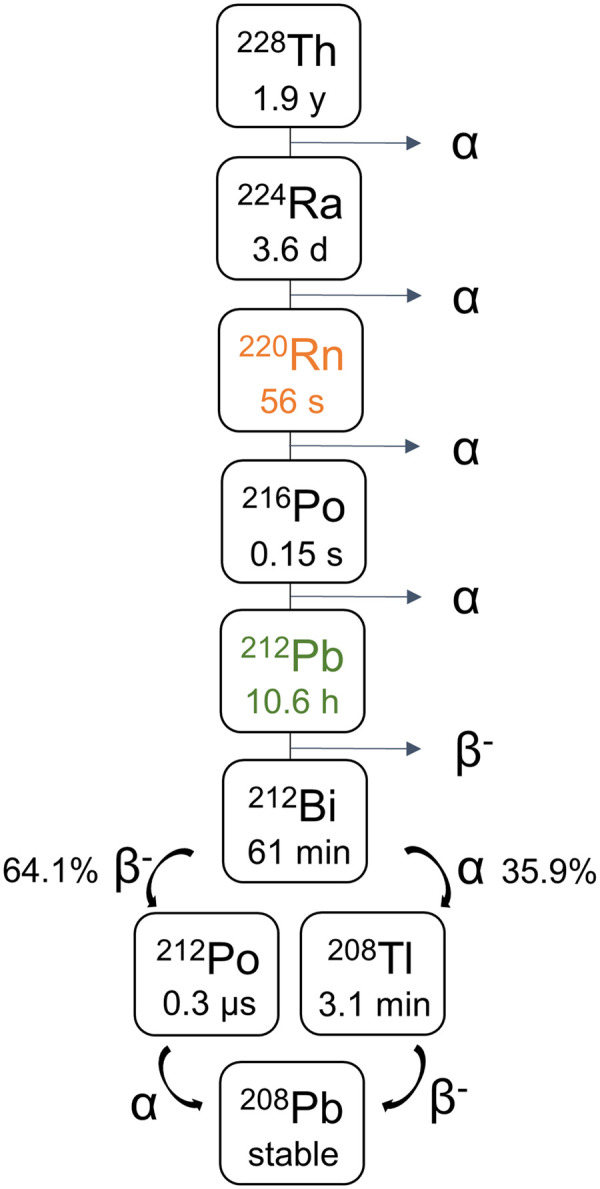
^228^Th series, with ^220^Rn and ^212^Pb highlighted.

A second approach is based on radon emanation, which involves obtaining ^212^Pb from gaseous ^220^Rn (half-life, 55.6 s) emanated from the decaying (^228^Th/)^224^Ra parent. Thus, ^212^Pb can be isolated from parent nuclides without the need for dedicated equipment for the separation process. Hassfjell and Hoff reported a generator comprising a ^228^Th source distributed within barium stearate and stored in a housing chamber connected to a vacuum pump (8). The source could be slid into the collection chamber via a gate valve. The generator experienced a relatively poor yield (11%–50%) because of radiation damage of the source when 40–50 MBq of ^228^Th were used. Other examples are based on 2-compartment systems in which ^220^Rn is transferred from a source chamber with parent nuclides into a collector chamber by airflow (9*–*11). These generators require significant effort and advanced equipment and have been tested in only small-scale production (≤2 MBq). Another drawback of such 2-compartment systems is that ^220^Rn may decay before reaching the collector chamber, potentially resulting in low ^212^Pb yields (9).

Here, we report a novel single-chamber generator based on ^220^Rn emanation from decaying ^224^Ra or ^228^Th to produce high yields of ^212^Pb for radiolabeling of ligands and monoclonal antibodies (mAbs). The generator is compact and user-friendly—key considerations for a shippable device that can be operated by the staff at a nuclear medicine facility.

## MATERIALS AND METHODS

### The ^228^Th/^224^Ra/^212^Pb Generator

**FIGURE 2.**
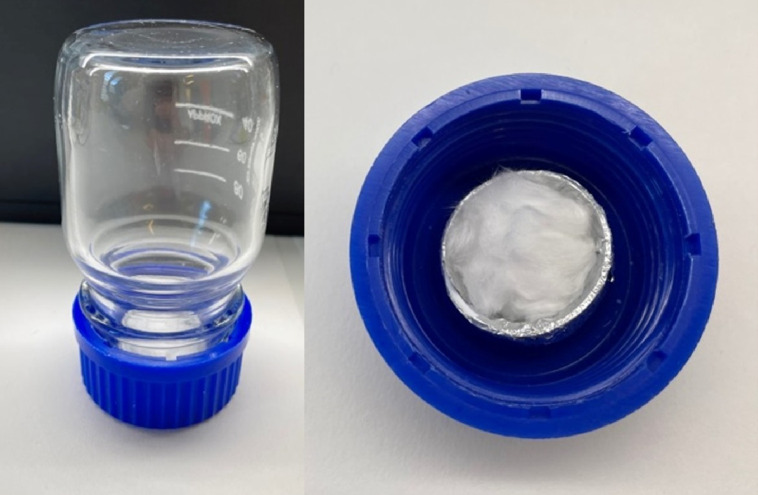
Single-chamber ^212^Pb generator consisting of glass flask and removable cap that contains ^228^Th or ^224^Ra source fixed onto porous quartz wool.

An earlier generation of the generator was previously reported (12), but the recent version was optimized to increase output capacity and reduce the risk of cross-contamination. The generator, consisting of a 100-mL glass flask standing upside down, with the radionuclide source contained in the screw cap (Fig. 2) (13), was kept at room temperature the entire time. ^228^Th (Eckert and Ziegler or Oak Ridge National Laboratory) or ^224^Ra (prepared as previously described (7)) in 100–200 μL of 0.1–1 M HCl was applied to approximately 0.2 g of porous quartz wool (ProQuarz GmbH). The quartz wool was placed on a small plastic cap covered in aluminum foil to minimize ^220^Rn retention and secured inside the screw cap (Fig. 2). During ^228^Th/^224^Ra decay, the short-lived ^220^Rn emanated from the quartz wool, followed by adsorption of the longer-lived ^212^Pb daughter onto the interior surfaces of the flask. After approximately 2 d, the flask was carefully replaced with a clean flask to harvest ^212^Pb and reuse the generator, ensuring no cross-contamination from the source. To extract the ^212^Pb, 0.5–1 mL of 0.1 M HCl solution was added, and the flask was carefully swirled to cover the inner surface for about 5 min before the solution was collected.

### Radioactivity Measurements

A pure source of ^224^Ra reaches transient equilibrium with ^212^Pb after 2 d. We evaluated the yield of the ^224^Ra-based generator when ^212^Pb was harvested after 2–3 d, or as the average yield for the ^228^Th-based generator when 1 generator was used multiple times with at least a 2-d interval. The yield was defined as the percentage of ^212^Pb activity adsorbed to the flask relative to parent ^224^Ra or ^228^Th. The yield was also evaluated for generators that were milked for the second time. Radioactivity was quantified by a radioisotope dose calibrator (CRC-25R; Capintec Inc.) (12).

The breakthrough of ^224^Ra or ^228^Th in the washout solution at harvesting was quantified indirectly through the ^212^Pb activity of decayed samples—activity that was measured in the 60- to 110-keV window on a γ-counter (automatic γ-counter; Hidex Oy) (12). The details of the measurements and calculations are described in Supplemental Section 1 (supplemental materials are available at http://jnm.snmjournals.org).

^220^Rn emanation from the generator and the dose rate resulting from x-rays and γ-rays were evaluated for radiation safety purposes as described in detail in Supplemental Section 2.

### Radiolabeling

To evaluate the quality of the extracted ^212^Pb, the tumor-targeting ligand NG001 (PSMA617-TCMC TFA; MedKoo Biosciences Inc.) and the *S*-2-(4-Isothiocyanatobenzyl)-1,4,7,10-tetraaza-1,4,7,10-tetra(2-carbamoylmethyl)cyclododecane (TCMC)–conjugated cetuximab (Erbitux; Merck Group) and rituximab (MabThera; Roche) were radiolabeled and the radiochemical purity was measured as described in Supplemental Section 3.

## RESULTS

### Generator Yield, Performance, and Feasibility

The single-chamber ^212^Pb generator was easy to use and handle. The ^212^Pb solution could be extracted at regular intervals, and the generator cap could be transferred to a clean flask each time for reuse. Its small size allowed measurement in a standard ionization chamber dose calibrator. The yield was approximately 62% for the tested ^224^Ra/^212^Pb generators of 2–22 MBq, of which 87%–91% of the deposited ^212^Pb could be extracted with 0.5–1 mL of 0.1 M HCl (Table 1; Supplemental Table 1). The ^228^Th-based generator of approximately 3.5 MBq had a stable yield of 67%–70% (Table 1; Supplemental Table 1). Hence, approximately 262 MBq of ^224^Ra and 163 MBq of ^228^Th are necessary initially per 100 MBq of ^212^Pb to be obtained after 2 d.

**TABLE 1.** Data on Performance of Generators Based on ^220^Rn Emanation from Source of ^224^Ra or ^228^Th in Equilibrium with Its Daughters

Type of generator	Yield	Available ^212^Pb in washout	Radioactivity breakthrough of parent source nuclide
^224^Ra source	62% (56%*)	91%	0.02%^†^ (0.0004%–0.14%)
^228^Th source	68%^‡^	87%	0.0001%–0.005%^¶^

* Second-time use of generator (*n* = 3).

^†^ Average of 9.

^‡^ Average of multiple uses of single generator (*n* = 8).

^¶^ In 6/8 samples, measured radioactivity was below quantification limit after >2 mo (<0.0015% breakthrough; Supplemental Section 1).

The breakthroughs of ^224^Ra and ^228^Th were attributed to cross-contamination from the source. The radioactivity of ^224^Ra and ^228^Th was 0.0004%–0.14% and 0.0001%–0.005% relative to ^212^Pb, respectively, at the time of harvesting ^212^Pb (Table 1; Supplemental Table 1). In 6 of 8 samples (65–269 kBq of ^212^Pb initially) from the ^228^Th-based generator, the measured radioactivity was below the quantification limit of the instrument.

### Radiation Safety Aspects

Our evaluation of generator integrity did not indicate any escape of ^220^Rn when the generator was closed. However, radon exposure from the generator is a potential radiation safety concern when the generator is opened, because the half-life of ^220^Rn is long enough for the gas to reach its surroundings. In the experimental setup in which a 1-MBq ^224^Ra-based generator was opened inside a sealed bag for 10 s, approximately 11% of the available ^220^Rn escaped (Supplemental Section 2).

Exposure to x-rays and γ-rays is another potential safety concern. The measurements on the surface of a 2-cm lead shield showed an average dose rate of 20 μSv/h per MBq of ^228^Th. The dose rate was considerably reduced to 2.3 μSv/h per MBq for a 5-cm lead shield and to 0.7 μSv/h per MBq for a 7-cm lead shield.

### Radiochemical Purity of Radioconjugates

The ^212^Pb extracted from ^224^Ra-based generators was used to radiolabel TCMC-conjugated ligands and mAbs with a high and reproducible radiochemical purity for all tested compounds (Table 2).

**TABLE 2.** Radiochemical Purity of Various Radioconjugates After Radiolabeling with ^212^Pb

Radiolabeled substance	Radiochemical purity (average ± SD)
^212^Pb-NG001 (*n* = 11)	97% ± 2%
^212^Pb-TCMC-cetuximab (*n* = 7)	99% ± 1%
^212^Pb-TCMC-rituximab (*n* = 6)	99% ± 1%

## DISCUSSION

Here, we present an experimental ^212^Pb generator that is compact, easy to use, and operable without advanced equipment or hazardous chemicals. These considerations are important for the convenient and efficient routine production of ^212^Pb in clinical applications. To our knowledge, there are no existing ^212^Pb generators that meet these criteria entirely (1*,*^14^*,*^15^). The ^228^Th-based generator bypasses the ^224^Ra separation step from ^228^Th while being a longer-lived device that facilitates upscaled production of ^212^Pb at an industrial scale. Results show that a single ^228^Th-based generator could be milked every 2–5 d to routinely supply high-purity ^212^Pb for research and development. Radiopharmacies and hospitals must consider the exemption limit of ^228^Th—which is a tenth of that of ^224^Ra and ^212^Pb in the European Union and United States—when applying for permits for certified use. No well-defined criteria for an acceptable level of ^228^Th impurity in a radiopharmaceutical exist, but for 7 of 8 samples, the values were below the acceptance limit (<0.002%) for the impurity level of another therapeutic radiopharmaceutical that is described in the *European Pharmacopoeia* (16). Assuming a 100-MBq patient dose, the value is comparable to the effective dose-derived annual limit of intake of ^228^Th (17). The breakthrough of ^224^Ra from the ^224^Ra-based generator was comparable to the current state of the art (14). Hence, a clinically relevant purity is achievable with the presented technology.

**FIGURE 3.**
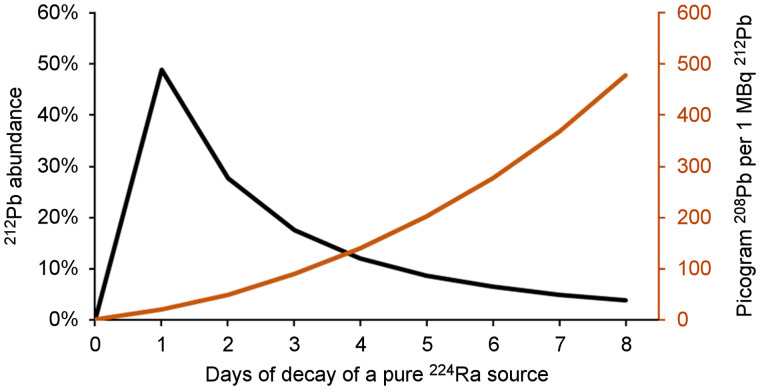
Relationship between radioactive ^212^Pb and stable daughter nuclide ^208^Pb as function of time, shown as abundance of ^212^Pb relative to total amount of lead (black curve) and mass concentration of ^208^Pb per 1 MBq of extracted ^212^Pb (red curve), assuming generator yield of 62%.

Decay of ^228^Th/^224^Ra results in an increasing accumulation of the stable daughter nuclide ^208^Pb in the generator, which potentially competes with ^212^Pb in radiolabeling procedures. The cumulative amount of ^208^Pb in the extracted ^212^Pb can be estimated on the basis of the generator yield (Fig. 3; Supplemental Section 4). In terms of mAb binding, these fractions may not influence radiochemical purity, as only 1 in about 2,000 mAbs needs to be bound by a ^212^Pb atom for a clinically relevant specific activity (18).

The current generator is a prototype from which a limited number of ^212^Pb extractions have been performed. Along with upscaling, the radiation safety and yield may be areas for improvement in future studies. Both issues can be addressed by design considerations. The source-holding material, its size and volume, and the inner surface area of the generator can be optimized to increase the levels of ^212^Pb depositing onto the surface. It should be verified that the holding material is not affected when one is working with higher radioactivity levels. A closed system with an integrated shielding unit for the source in which the source or the shielding unit is movable (e.g., by a plunger) would facilitate operation without exposing the source during ^212^Pb extraction. Handling the generator inside hot cells or inside glove boxes or bags, or the use of tongs or similar equipment to protect the operator, is an important measure when working with clinically relevant activity levels (e.g., 100 MBq). Automation of the extraction process is considered feasible given that it entails only a surface-washing step and subsequent recovery of the solution.

## CONCLUSION

^220^Rn emanation can be exploited to create a simple and effective generator that produces high-purity ^212^Pb without the need for advanced equipment, labor-intensive steps, or hazardous chemicals. Future versions of the presented technology should include simple modifications to shield the source during extraction of the ^212^Pb. The generator represents a promising method for efficient ^212^Pb production.

## DISCLOSURE

Sciencons AS, owned by Roy Larsen, holds intellectual property rights for the presented technology under a patent application. Ruth Li and Vilde Stenberg were industrial PhD students financially supported by the Norwegian Research Council (grants 291228 and 290639) at the time of contributing to the article, at which Vilde Stenberg was also a shareholder at ArtBio AS. Ruth Li is employed at Oncoinvent AS, Vilde Stenberg is employed at ArtBio AS, and Roy Larsen is the chairman of the board of both companies, which use the presented technology for research-and-development projects. Roy Larsen owns stock directly or indirectly in Sciencons AS, Oncoinvent AS, and ArtBio AS. No other potential conflict of interest relevant to this article was reported.

## Notes

KEY POINTS **QUESTION:** Can ^212^Pb, intended for radiopharmaceuticals, be produced by a simple generator based on ^220^Rn emanation from a ^228^Th or ^224^Ra source? **PERTINENT FINDINGS:** The proposed generator was easy to handle and could routinely be used to produce ^212^Pb of high purity, suitable for radiolabeling of antibodies and ligands. **IMPLICATIONS FOR PATIENT CARE:** Rapid and efficient production methods such as the one proposed are important for ^212^Pb to be available for patients with metastatic cancer.
